# From classical Chinese formula to modern mechanism: how Xiao-Yao-San modulates key signaling pathways in depression

**DOI:** 10.1186/s13020-025-01315-7

**Published:** 2026-01-15

**Authors:** Caiyan Qu, Rongyanqi Wang, Aiai Liu, Yueyun Liu, Zhentao Zhao, Wenzhi Hao, Jiaxu Chen

**Affiliations:** 1 School of Traditional Chinese Medicine, Beijing University of Chinese Medicine, Beijing, China; 2 Guangzhou Key Laboratory of Formula-Pattern of Traditional Chinese Medicine, School of Traditional Chinese Medicine, Jinan University, Guangzhou, China

**Keywords:** Xiao-Yao-San, Depression, Signaling pathway, Pi3k/akt, NF-KB, NLRP3

## Abstract

Depression is a widespread mental disorder with profound effects on both physical and psychological health. Aberrant signal transduction contributes to depression by impairing neuronal function, reducing synaptic plasticity, and disrupting neurotransmitter transmission. Aberrant signal transduction can impair neuronal function, reduce synaptic plasticity, and disrupt neurotransmitter transmission, thereby contributing to the development of depression. Xiao-Yao-San (XYS), a traditional Chinese medicine (TCM) formula, has been extensively employed in the treatment of depression, with a broad therapeutic profile. This review aims to critically assess current scientific evidence on the antidepressant effects of XYS, focusing on its modulation of key signaling pathways. The goal is to provide a more robust mechanistic foundation for XYS-based therapies and offer insights for developing novel antidepressants that target signaling pathways. We systematically searched PubMed, Web of Science, ScienceDirect, CNKI, and Wanfang databases from inception to May 1, 2025, using relevant terms to identify studies on XYS and its active components, particularly those elucidating its regulation of signaling pathways involved in depression. XYS and its active ingredients modulate several crucial signaling pathways implicated in depression, including the phosphatidylinositol 3-kinase/protein kinase B (PI3K/Akt), NOD-like receptor family pyrin domain containing 3 (NLRP3), nuclear factor-kappa B (NF-κB), and mitogen-activated protein kinase (MAPK) pathways. The antidepressant effects of XYS are primarily mediated through anti-inflammatory actions, improved hippocampal architecture, suppression of neuronal and mitochondrial apoptosis, reduction of oxidative stress, and enhancement of synaptic plasticity. Despite current limitations, this review identifies future research directions focusing on unexplored and cross-linked signalling pathways, which may support the development of signalling-targeted antidepressant agents based on XYS.

**Supplementary Information:**

The online version contains supplementary material available at 10.1186/s13020-025-01315-7.

## Introduction

Depression is a common and disabling psychiatric disorder, characterized by persistent low mood, anhedonia, and social withdrawal, often accompanied by cognitive impairment, sleep disturbances, appetite changes, weight fluctuation, and suicidal ideation [1]. According to the World Health Organization, mental disorders affect approximately 13% of the global population, with depression accounting for 28.9% of cases. Its high recurrence, underdiagnosis, and substantial psychosomatic burden place depression among the leading contributors to global disease burden[2].

**Fig. 1:**
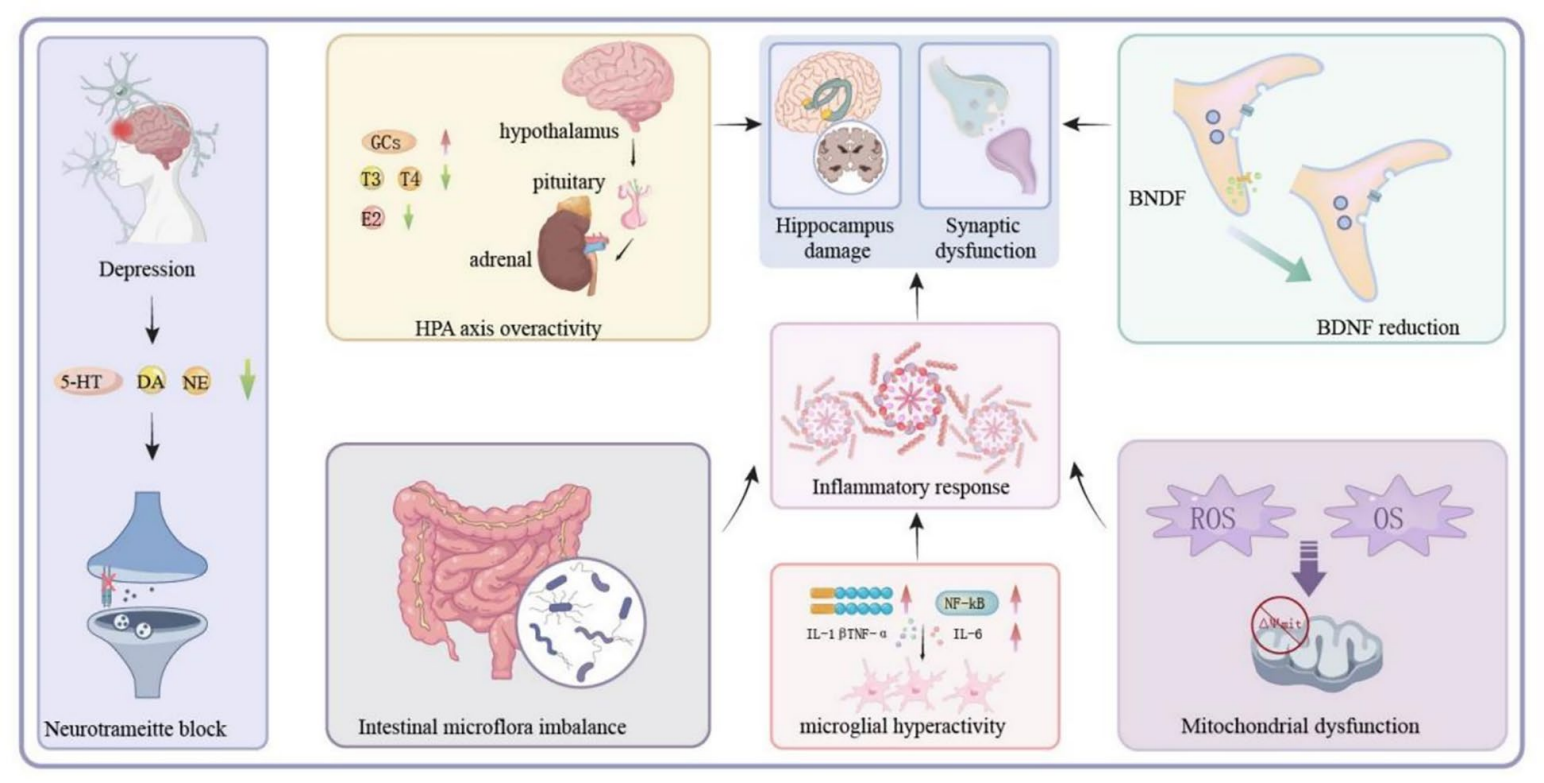
The pathogenesis of depression

The pathophysiology of depression is multifactorial and not yet fully elucidated. Key mechanisms include monoamine neurotransmitter imbalance [3], neuroinflammation [3], dysbiosis of the gut microbiota [4], hyperactivity of the hypothalamic pituitary adrenal (HPA) axis[5], alterations in brain-derived neurotrophic factor(BDNF) level [6], and oxidative stress [7] (Fig. 1). Signal transduction pathways, which mediate intracellular and intercellular communication via ligand-receptor interactions and downstream cascades, are integral to these processes [8]. Disruptions in these pathways may result in neuronal dysfunction, impaired synaptic plasticity, and altered neurotransmitter signaling, all of which contribute to depressive symptoms [9].

For instance, depression is associated with diminished levels of serotonin (5-HT), norepinephrine (NE), and dopamine in the brain [10]. The cAMP/CREB signaling axis is known to enhance the synthesis of 5-HT and NE, thereby exerting an antidepressant effect [11]. Elevated pro-inflammatory cytokines, including tumor necrosis factor-α (TNF-α), interleukin-1β (IL-1β), and interleukin-6 (IL-6), have been observed in both patients and animal models of depression [12]. The activation of NLRP3 inflammasomes and NF-κB pathways intensifies neuroinflammation and aggravates depressive symptoms [13]. Overactivation of microglia further exacerbates inflammation, leading to neuronal injury and synaptic disruption [14].

Additionally, chronic HPA axis hyperactivity leads to excessive glucocorticoid production, resulting in hippocampal atrophy, impaired neurogenesis, and reduced synaptic plasticity [5]. BDNF, essential for neuronal survival and synaptic modulation, is consistently downregulated in depression, contributing to decreased neuroplasticity [6]. The MAPK and PI3K/Akt pathways are involved in promoting neuronal survival and are implicated in antidepressant mechanisms [15]. Moreover, the mammalian target of rapamycin (mTOR) integrates nutrient and energy signals, regulating mitochondrial autophagy and cellular metabolism. In depression, increased reactive oxygen species (ROS) and decreased antioxidant activity disrupt mitochondrial function, exacerbate neuronal apoptosis, and provoke inflammatory responses [16]. Collectively, these disrupted signalling pathways represent central nodes in the pathogenesis of depression and are attractive targets for therapeutic intervention.

Despite this understanding, clinical treatment of depression remains challenging. Selective serotonin reuptake inhibitors (SSRIs), such as fluoxetine and paroxetine [17], are first-line therapies but are limited by their single-target mechanisms, delayed onset, and frequent side effects, resulting in poor adherence and suboptimal outcomes [18]. In contrast, traditional Chinese medicine (TCM) has gained recognition for its effectiveness in managing mood disorders [19]. The possible pathogenesis of depression is shown in Fig. 1

Xiao-Yao-San (XYS), a classical TCM formula first recorded in the *Taiping Huimin Heji Ju Fang* during the Song Dynasty, is one such remedy with demonstrated antidepressant effects. XYS, also known as Xiaoyao Pills (XYW), is composed of *Bupleuri Radix*, *Rhizoma Atractylodis Macrocephalae*, *Herba Menthae*, *Radix Angelicae Sinensis*, *Radix Glycyrrhizae*, *Radix Scutellariae*, *Radix Paeoniae Alba*, and ginger, in a traditional ratio of 2:2:2:2:2:2:1. Pharmacological studies show that XYS modulates monoaminergic systems[20], regulates the HPA axis [21], reduces neuroinflammation [22], and ameliorates structural alterations in the amygdala and hypothalamus associated with depression [23].

Furthermore, component profiling using UPLC-Q-TOF–MS/MS has identified 102 chemical constituents, 10 prototypical compounds, and 16 brain-penetrating metabolites within XYS. Notably, paeoniflorin, saikosaponin D, and glycyrrhizin exhibit high binding affinities to depression-related targets[24]. Recent investigations have shown how these active components exert antidepressant effects by regulating signalling pathways. While these studies affirm the therapeutic relevance of XYS, mechanistic details remain to be fully delineated, and the development of XYS-derived pharmaceuticals continues to face obstacles.

**Fig. 2:**
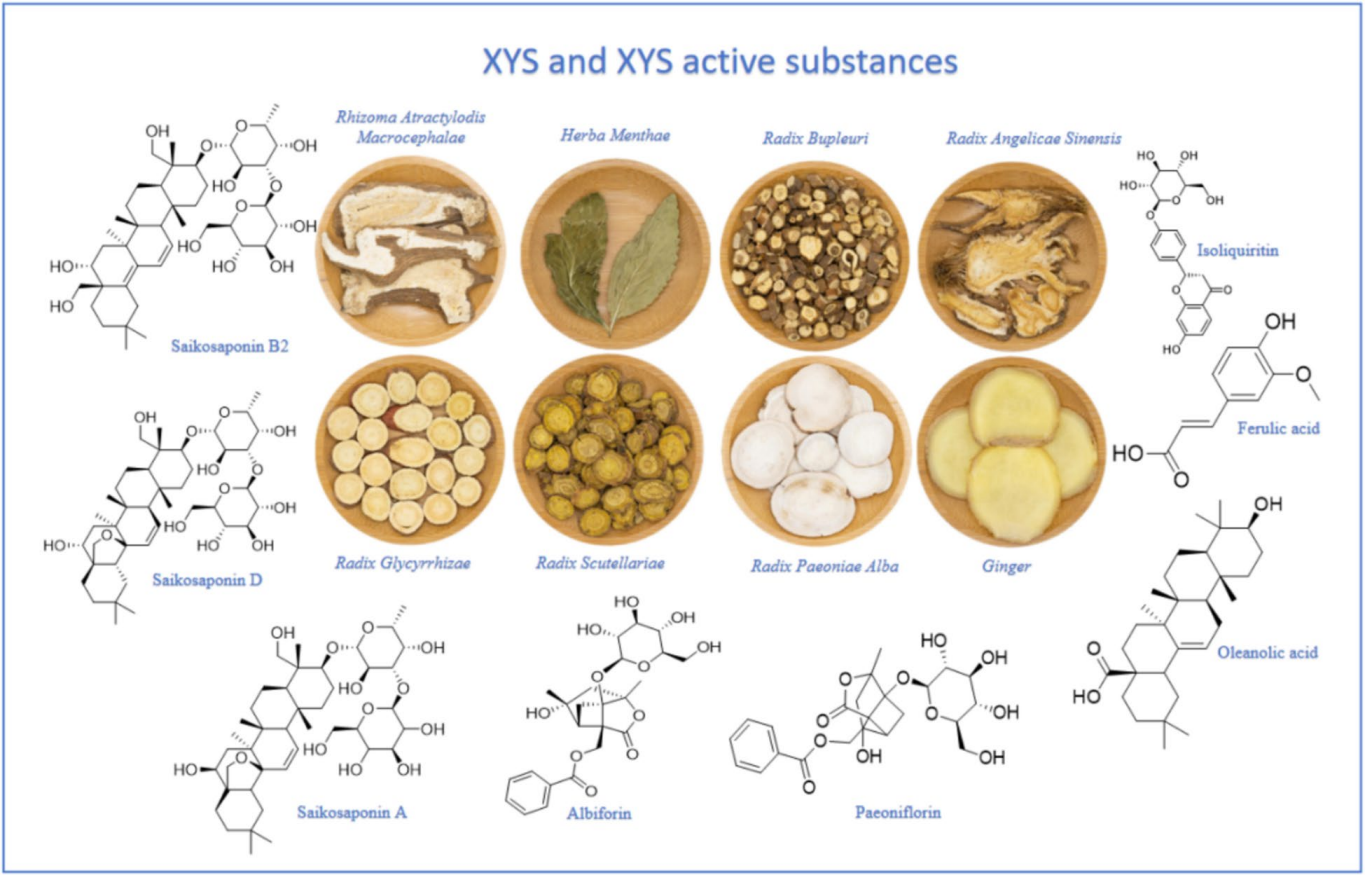
XYS components and XYS active substances

Accordingly, this review synthesises existing research on XYS and its bioactive constituents in modulating depression-related signalling pathways. In doing so, it aims to consolidate the mechanistic basis for XYS's clinical application and inform future drug development targeting these pathways. The components of XYS and XYS active substances are shown in Fig. 2

## Materials and methods

### Search strategy

**Fig. 3:**
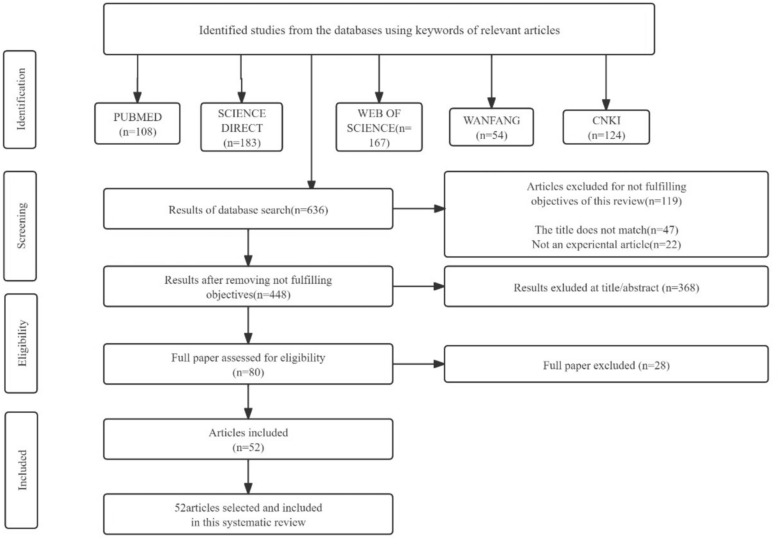
Literatures search process

A comprehensive literature search was conducted using PubMed, Web of Science, ScienceDirect, CNKI, and Wanfang databases to identify studies examining the effects of XYS and its active constituents on depression-related signaling pathways. Searches covered all publications from database inception through May 1, 2025. Search terms included combinations of "Xiaoyaosan," "Xiaoyao," "active ingredient of Xiaoyaosan," "depression," "anxiety," and "signaling pathway." The detailed search process is illustrated in Fig. 3**.**

### Inclusion and exclusion criteria and data extraction

Inclusion criteria were: (1) in vivo or in vitro studies investigating the modulation of signaling pathways by XYS or its active constituents in models of depression; (2) interventions using the complete XYS prescription (comprising the eight traditional herbs) or its individual active compounds, with no restrictions on dosage, frequency, or route of administration; (3) no limitations regarding animal species, sex, age, weight, or cell type; and (4) a focus on signaling pathways relevant to depression.

Exclusion criteria included: (1) studies with incomplete or inaccurate outcome data; (2) duplicate publications; (3) studies in which XYS or its components were used only as adjunct therapies or modified formulas not strictly composed of the traditional eight herbs; and (4) studies that did not examine signaling pathways in the context of depression.

Full texts of eligible studies were reviewed, and key information was extracted, including: model type and species, intervention details, dosage, sample sizes, treatment duration, outcome measures, involved signaling pathways, and proposed mechanisms of action.

## Evidence supporting the use of XYS in depression

### Clinical studies

Clinically, XYS is administered alone, symptomatically modified, or combined with conventional treatments. Its formulations include decoctions, pills, and powders. Regardless of form or combination, XYS consistently demonstrates therapeutic efficacy and safety in treating depression.

Recent randomized controlled trials (RCTs) have further consolidated the evidence regarding the efficacy of XYS and its modified formulations in the management of depression-related disorders. Multiple RCTs have demonstrated that XYS—whether administered as monotherapy or adjuvant therapy—significantly reduces depression scores on validated assessment scales, including the Hamilton Depression Rating Scale (HAMD), Hamilton Rating Scale for Depression (HRSD), Beck Depression Inventory (BDI), and Self-Rating Depression Scale (SDS), among patients with mild-to-moderate depression[25, 26], perimenopausal depression[27], burning mouth syndrome[28], premenstrual syndrome[29], functional dyspepsia[30], or mixed anxiety-depressive disorder (MADD)[31]. Several of these studies have also reported higher response rates with XYS, highlighting its superiority over placebo or monotherapy with conventional Western antidepressants. Nevertheless, XYS has failed to yield significant improvements in mood or depressive symptoms in individuals with post-coronavirus disease 2019 (post-COVID-19) depression or functional dyspepsia complicated by mild depression[32]. Additionally, emerging evidence suggests that combining MDXYS with selective serotonin reuptake inhibitors (SSRIs; e.g., escitalopram, sertraline) may potentiate antidepressant effects[33], with particular efficacy observed in patients with post-stroke depression (PSD) [34]and major depressive disorder (MDD). Another study demonstrated that XYS restored serum metabolite levels—such as alanine, choline, trimethylamine-N-oxide, glutamine, lactate, and glucose—in depressed patients to near-normal values when compared to healthy controls [35]. The specific clinical research results are shown in Table 1.

**Table 1:** Summary of clinical results of XYS treatment for depression

Research type	Diagnosis	Participants control/experimental	Intervention control/experimental	period	Main outcome	Safety	References
Randomized, double-blind, placebo-controlled clinical trial	Mild to moderate depression	20(12MDD-mild,4MDD-severe,age < 45)/21(12 MDD-mild,3MDD-severe,age < 45),10health	placebo 8 pills tid/XYW 8 pills tid	8 weeks	HAMD-17 ↓ at 6 week (p < 0.0001); largest gain in psychomotor retardation (p < 0.05). Rebalanced gene expression (38%↓, 20%↑) and reversed DNA hypomethylation at RTN4/FFAR2	Not explicitly mentioned	[25]
Randomized,double-blind,double-dummy,multicenter,parallel-controlled clinical trial	Mild to moderate major depression	96 (29 males, 67 females; mean age 44 ± 13 years; baseline HAMD score 25 ± 5, HAMA score 17 ± 6)/95 (27 males, 68 females; mean age 42 ± 12 years; baseline HAMD score 25 ± 4, HAMA score 16 ± 5)	Sertraline 50-100 mg qd + MDXY placebo/MDXY capsule 10 g bid + sertraline placebo	8 weeks	At week 8, the HAMD response rate was 63.16% in the experimental group versus 53.13% in the control group;The experimental group showed significantly greater HAMA score reductions at week 2 (3.4 ± 3.5 vs 2.9 ± 4.5) and week 12 (10.6 ± 5.5 vs 8.8 ± 6.2; both P < 0.05);Significant advantages were also observed for sleep disturbance, somatic anxiety, and CGI-EI scores at weeks 8 and 12 (all P < 0.05)	AE incidence at week 12: 3.16% (experimental) vs 1.04% (control), P > 0.05 Common AEs in the experimental group: dry mouth 10.53%, headache 14.74%, sweating 11.58%, nausea 4.21%, dizziness 1.05%; no insomnia or hypotension reported	[26]
Randomized, double-blind, placebo-controlled clinical trial	Functional dyspepsia with comorbid mild depression in perimenopausal women	90 (age 42–52 years, mean 46.09 ± 2.45 years; disease course 1–9 years, mean 3.96 ± 2.43 years; baseline HRSD score 12.12 ± 2.29)/90 (age 41–49 years, mean 45.30 ± 2.81 years; disease course 1–8 years, mean 4.20 ± 2.30 years; baseline HRSD score 11.44 ± 2.15)	Placebo3g bid/XYW 3 g bid	8 weeks	HRSD score: After 8 weeks, the experimental group (6.20 ± 2.08) was significantly lower than the control group (9.14 ± 1.67) (P < 0.01)	No adverse drug reactions reported in both groups during treatment and follow-up	[27]
Open-label,randomized controlled trial	Burning mouth syndromewith comorbid depressive symptoms)	39(1 male,38female,age 31–79, mean 57.01 ± 10.27;baseline BDI score 14.38 ± 10.21)/39(2 males, 37females, age 29–78, mean 55.39 ± 9.63; baseline BDI score 12.76 ± 8.69)	Methylcobalamin tablets 0.5 mg tid/MDXY tablets 2.8 g bid + Methylcobalamin tablets 0.5 mg tid	6 weeks	The BDI of the experimental group significantly improved at 2 weeks (6.13 ± 5.39 vs 9.65 ± 5.86), 4 weeks (5.95 ± 7.91 vs 10.06 ± 7.46), and 6 weeks (3.79 ± 4.27 vs 8.24 ± 6.57), all P < 0.05 compared with control group.No significant BAI different (P > 0.05);	No adverse effects occurred in either group	[28]
Multi-center,randomized,double-blind,placebo-controlled trial	Premenstrual syndrome with depression	69(age 29.22 ± 6.11;baseline TCMSSS depressive symptom score:0points 13cases,2points 35cases,4points 20cases,6points 1case)/67(age 29.47 ± 5.58;baseline TCMSSS depressive symptom score: 0points 8cases,2points 39cases,4points 20cases,6points 0cases)	Placebo 6 g bid/MDXY 6 g bid	3menstrual cycles	DRSP reduction:experimental group > control group (P < 0.001); TCMSSS response rate:70.15% vs. 46.38%(P < 0.05); depression scores continued to decline through cycle3and remained superior to placebo	AE rates, vital signs and labs were comparable between groups; one SAE (abdominal pain) in the active arm was deemed unrelated to treatment	[29]
Multicenter,randomized,double-blind,placebo-controlled clinical trial	Functional dyspepsia with comorbid mild depressive symptoms	71(17 males,54 females;mean age 43.0 ± 12.1 years;baseline HAMD score 10.9 ± 2.4,HAMA score 9.5 ± 3.8)/70(17males,53females;mean age45.9 ± 13.6 years;baseline HAMD score 11.1 ± 2.5,HAMA score9.7 ± 3.5)	Placebo 6 g bid/MDXY 6 g bid	4 weeks	HAMD score: The experimental group showed greater improvement than the control group at 4 and 8 weeks (4 weeks: P = 0.093; 8 weeks: P = 0.174)	AEs: 30 in the treatment group vs 20 in the control, P = 0.240; drug-related AEs were three mild cases (diarrhea, constipation, elevated liver enzymes, one each). One SAE (ocular tumor) was unrelated to the study drug	[30]
Multicenter,randomized,double-blind,placebo-controlled trial	Mixed Anxiety-Depressive Disorder	191(33 males, 158 females;mean age 45.20 ± 11.77 years; baseline HAMD-17 score 11.36 ± 3.07, SDS score 51.31 ± 9.48)/194 (29 males, 165 females;mean age 43.68 ± 11.07 years;baseline HAMD-17 score 11.36 ± 3.20, SDS score 51.33 ± 9.21)	Placebo granule, 1 bag bid/MDXY granule,3 g bid	8 weeks	Compared with controls, the intervention group exhibited lower HAMD-17scores at 4 week (7.30 ± 3.53 vs 8.50 ± 3.33) and 8 week (4.37 ± 3.43 vs 6.70 ± 3.64; both P < 0.0001), and lower SDS scores at 4 wk (44.43 ± 8.30 vs 46.52 ± 8.30; P < 0.05) and 8 wk (39.71 ± 8.21 vs 43.40 ± 8.63; P < 0.0001) Remission rates (HAMD-17 ≤ 7) rose from 44.31% to 80.65% in the intervention group versus 31.58% to58.68% in controls (P < 0.05) The TCM “depression” symptom reduction rate at 8 wk was 58.39% versus 28.83% (P < 0.0001)	There was no difference in AE/SAE between the two groups, and there were no clinical abnormalities in vital signs and laboratory indicators	[31]
Randomized,double-blind,placebo-controlled trial	COVID-19 recovery phase with depression	95(33males,63females;median age 54 years;baseline VAS scores:irritability 4.4 ± 2.1,anxiety 4.6 ± 1.7,poor sleep5.0 ± 1.7)/100(35males,65females;median age54 years;baseline VAS scores:irritability 4.4 ± 2.2,anxiety 4.9 ± 1.8,poor sleep 5.5 ± 1.7)	Placebo XYcapsule 4 capsules bid/XYcapsule 4 capsules bid	2 weeks	No statistically significant amelioration of sleep or mood disturbances was observed in COVID-19 convalescents (all P > 0.05)	AE rate 2.1% (2/95) vs 1% (1/100), P > 0.05; no drug-related vital-sign anomalies or serious adverse events reported	[32]
Double-blind,randomized,placebo-controlled clinical trial	Depressive disorder	15 (age 18–60 years; median age 33.93 ± 13.88 years; disease course median 24 days; 9 males, 6 females)/17 (age 18–60 years; median age 25 (17–58) years; disease course median 13.5 days; 9 males, 8 females)	Placebo + SSRIs/MDXY 1 bag tid + SSRIs	4 weeks	HAMD-24 score: The factor V (delayed symptoms) score (P = 0.033) and item 1 (depressive mood) score (P = 0.024) of the experimental group were significantly lower than those of the control group	No serious adverse events or drug-related safety risks explicitly mentioned	[33]
Prospective,randomized,double-blind,parallel controlled clinical trial	Post-stroke depression	57 (age 18–75 years; no antidepressant use in previous 2 weeks)/57 (age 18–75 years; no antidepressant use in previous 2 weeks)	MDXYS simulant 1 bag tid + Escitalopram oxalate 5 mg bid/MDXY 1 bag tid + Escitalopram oxalate 5 mg bid	8 weeks	HAMD score reduction rate: expected to be 85% in the experimental group and 68% in the control group	No obvious abnormalities or adverse reactions were observed	[34]
Prospective, randomized, double-blind, parallel controlled clinical trial	Depression	16 healthy volunteers (10males, 6females,age 44.6 ± 7.0 years, HAMD score ≤ 7)/16 depressed patients (10males, 6females, age 49.6 ± 6.8 years, baseline HAMD score 20.7 ± 2.1, CGI-SI score 3.8 ± 0.4)	no/XYS 1 dose/day	8 weeks	HAMD score significantly decreased to 4.9 ± 5.0 (P < 0.01); CGI-SI score reduced to 1.2 ± 0.5 (P < 0.01) Plasma metabolites (alanine, choline, TMAO, glutamine, lactate, glucose) reversed to normal levels Combination of lactate, TMAO and phenylalanine showed high diagnostic AUC (0.953)	Not explicitly mentioned	[35]

Several meta-analyses support these positive findings from clinical trials. A systematic review and meta-analysis of 26 randomized controlled trials (RCTs) involving 1,837 patients found that XYS monotherapy had a lower incidence of adverse effects compared to conventional antidepressants (amitriptyline, venlafaxine, and fluoxetine), with comparable improvements in depression scale scores [36]Another meta-analysis of seven studies involving 607 patients with post-stroke depression demonstrated that XYS adjunctive therapy increased overall treatment response and decreased depression severity scores, with no serious adverse events reported[37]. A comprehensive network meta-analysis that included 198 RCTs covering 17 Chinese herbal formulas and 8,923 patients ranked XYS among the top three most effective treatments in reducing Hamilton Depression scores, and it had the lowest rate of adverse effects among all treatments assessed[38]. Additionally, a meta-analysis of 48 RCTs evaluating treatments for functional gastrointestinal disorders (FGIDs), XYS significantly reduced symptom severity and relapse rates [39]. Given the known comorbidity between FGIDs and depression [40], these findings suggest that XYS may exert beneficial effects on depressive symptoms through the gut-brain axis.

Collectively, these clinical findings underscore the therapeutic potential of XYS in treating depression, both through symptomatic relief and by improving underlying molecular and metabolic disturbances, all while exhibiting a favorable safety profile. However, future high-quality RCTs with larger sample sizes, more extended follow-up periods, and rigorous methodology are needed to confirm these findings and address existing limitations.

### Systems biology of XYS for the treatment of depression

XYS, a classical Chinese herbal formula, has long been used in clinical practice for treating depression, with increasing experimental evidence supporting its efficacy. However, the underlying mechanisms remain incompletely understood due to the complex, multi-component, multi-target, and multi-pathway nature of the system. Emerging approaches such as network pharmacology, integrated multi-omics, and molecular validation provide valuable tools for identifying bioactive components, disease-associated targets, and therapeutic mechanisms in TCM.

Network pharmacology integrates drug-target and disease-target networks to predict potential molecular targets and signaling pathways, revealing the system-level actions of XYS. For instance, based on network pharmacology and target-pathway enrichment analysis, Zhang et al. constructed a "target–pathway–target" interaction map, identifying Protein Kinase B alpha (AKT1), Phosphoinositide-3-Kinase Regulatory Subunit 1 (PIK3R1), Nuclear Factor Kappa-B Subunit 1 (NFKB1), and RelA (p65) as core targets, which are linked to neurological diseases including depression[41]. Another study reported 68 core depression-related targets of XYS and found significant enrichment in the PI3K-Akt and MAPK signaling pathways. Gene Ontology (GO) analysis further indicated associations with apoptosis and inflammatory responses[24]. Similarly, additional network pharmacology analyses revealed overlapping targets, such as IL-1β, IL-6, and TNF, with enriched signaling pathways including the Advanced Glycation End-products (AGE-RAGE) signaling pathway, Hypoxia-Inducible Factor 1 (HIF-1), and calcium signaling [42].

Integrating stable isotope-resolved metabolomics (SIRM), network pharmacology, and transmission electron microscopy (TEM), one study demonstrated that XYS alleviates hippocampal tricarboxylic acid (TCA) cycle dysfunction in chronic unpredictable mild stress (CUMS) rats by targeting enzymes such as ATP citrate lyase (ACLK), glutamate dehydrogenase (GLDH), glutamic-oxaloacetic transaminase (GOT), and pyruvate carboxylase (PC), with troxerutin identified as a key active compound [43].

Metabolomics, a core component of systems biology, reflects physiological status and bridges gene expression with phenotype. Using 16S rRNA sequencing and proton nuclear magnetic resonance (^1^H NMR), researchers found that XYS significantly modulated gut microbiota composition and normalized nine renal metabolites in CUMS rats [44]. Chen et al. employed ^1^H NMR, liquid chromatography–mass spectrometry (LC–MS), and commercial assays to demonstrate that XYS exerts antidepressant and hepatoprotective effects by modulating glutamate/glutamine metabolism, maintaining ammonia homeostasis, and enhancing energy metabolism [45].

Wu et al. injected ^13^C_6_-glucose via the tail vein and analyzed metabolite isotopic distributions using LC–MS in hippocampal tissues. The findings showed that XYS improved hippocampal glucose metabolism and mitochondrial function by acting on multiple targets and metabolic axes, including the pyruvate-lactate axis, PC, pyruvate dehydrogenase (PDH), succinate dehydrogenase (SDH), and mitochondrial respiratory chain complexes (MRCC-V) [46]. A multi-omics study incorporating metabolomics, microbiomics, and molecular docking identified fatty acid amide hydrolase (FAAH) as a primary target. Inhibition of FAAH increased endocannabinoid levels, thereby alleviating depressive symptoms[47].

Further LC–MS-based metabolomics analysis of liver mitochondria in CUMS rats identified 18 altered metabolites, of which nine were significantly regulated by XYS. These were enriched in six key pathways: phenylalanine metabolism, glutathione metabolism, alanine-aspartate-glutamate metabolism, the TCA cycle, glyoxylate and dicarboxylate metabolism, and pyrimidine metabolism[42].

Meng et al. utilized whole-transcriptome sequencing and bioinformatics to explore the antidepressant mechanisms of XYS. Their results revealed that XYS modulated the expression of miRNAs, lncRNAs, and circRNAs, thereby indirectly regulating downstream genes involved in synaptic function and neuronal activity. Bioinformatic analysis indicated that XYS modulated key synaptic signaling pathways, including the BDNF/TrkB/PI3K axis [48]. Wang et al. employed whole-genome bisulfite sequencing (WGBS) and RNA-seq to identify 14 differentially expressed genes through integrated methylation and transcriptome analysis. XYS treatment was associated with methylation changes in the Lim homeobox transcription factor 1 beta (*Lmx1b*), ATP-binding cassette Subfamily C member 5 (*Abcc5*), glypican-3 (*Gpc3*), and complement factor B (*Cfb*) genes, suggesting that epigenetic regulation of these genes contributes to its antidepressant effects [49].

In summary, the antidepressant mechanisms of XYS are multifaceted, involving metabolic regulation, modulation of signaling pathways, restoration of gut microbiota, transcriptomic adjustments, and epigenetic remodeling. These coordinated effects enhance neuronal function and energy metabolism, suppress inflammatory processes, and regulate neurotransmitter levels, thereby exerting comprehensive therapeutic benefits in the treatment of depression.

## XYS and XYS active substances regulate signaling pathways to improve depression.

### PI3K/Akt signaling pathway

The PI3K/Akt signaling pathway is a critical survival-promoting cascade activated by various growth factors, functioning as a key downstream effector of the BDNF/TrkB axis [15]. Extracellular stimuli like BDNF bind to receptors (e.g., TrkB), triggering dimerization and autophosphorylation, which initiates PI3K/Akt pathway activation [50]. This leads to the recruitment of PI3K to the cell membrane, where it catalyzes the conversion of phosphatidylinositol bisphosphate (PIP2) into phosphatidylinositol triphosphate (PIP3) [51]. PIP3 serves as a second messenger to recruit pleckstrin homology (PH) domain-containing proteins, notably Akt, to the membrane. Akt is then phosphorylated at Thr308 by phosphoinositide-dependent kinase 1 (PDK1) and at Ser473 by mTORC2, fully activating its kinase function [52, 53]. Akt activity is dependent on phosphorylation of Thr 308 and Ser 473 [54]. Akt subsequently regulates a variety of downstream targets, including glycogen synthase kinase 3 beta (GSK3β), mTOR, forkhead box O (FoxO), and bcl-2 antagonist of cell death (Bad), thereby modulating cell survival, metabolism, synaptic plasticity, and apoptosis [55, 56].

Enhancing PI3K/Akt signaling can attenuate depression through multiple mechanisms. Upregulation of this pathway has been shown to reduce GSK3β activity, thereby suppressing inflammatory cytokine production and mitigating neuroinflammation [56]. Antidepressants such as atorvastatin activate the PI3K/Akt/GSK3β pathway to improve mood, cognition, and hippocampal neurogenesis [57, 58]. Moreover, PI3K/Akt enhances synaptic function by promoting the expression of synaptic proteins, such as BDNF, PSD95, and synaptophysin, thereby regulating the glutamatergic system [59]. PI3K/Akt also regulates the function of the glutamatergic system[60], supporting the release of neurotrophic factors [61], maintaining mitochondrial function [62], and preventing hippocampal neuronal apoptosis [63]. Numerous studies confirm that XYS and its active components exert antidepressant effects by modulating the PI3K/Akt pathway.

#### Regulation of PI3K/Akt signaling by XYS

*Neuroprotection* Meng et al. conducted transcriptomic profiling to investigate the antidepressant mechanism of XYS, identifying 753 differentially expressed lncRNAs, 28 circRNAs, 101 miRNAs, and 477 mRNAs. Enrichment analysis indicated that XYS modulates synaptic function and ameliorates depression by targeting neurotrophic factor and PI3K/Akt signaling pathways. In vivo studies further confirmed that XYS activates the BDNF/TrkB/PI3K axis, increasing synaptic length, density, and complexity in the CA1 and CA3 hippocampal regions, as well as MAP2 expression [48]. Another study found that XYS counteracted CUMS-induced downregulation of glucocorticoid receptors (GR), phosphorylated GR (p-GR), connexin 43 (Cx43), and BDNF, suggesting that its antidepressant effect is mediated via the Cx43/GR/BDNF pathway[64]. Zhou et al. demonstrated that XYS reduced glutamate levels and serum corticosterone in CUMS rats, restored hippocampal neuron integrity, increased MAP2 and NR2B expression in the CA1 region, and upregulated hippocampal PI3K expression. XYS treatment also improved body weight and food intake, indicating that its therapeutic effects may involve modulation of NR2B and PI3K/Akt signaling to attenuate glutamate excitotoxicity[65]. Shan's study on vascular dementia indicated that XYS improved anxiety and depression-like behaviors by promoting myelin repair within the mPFC-BLA neural loop, enhancing inter-nuclear communication. These improvements were linked to the activation of the PI3K/Akt/mTOR pathway, with 10 g/kg and 20 g/kg doses showing stronger effects [66].

*Anti-oxidant potential* In olfactory bulbectomy (OB)-induced depression models, XYS reversed behavioral deficits and oxidative stress by activating the PI3K/Akt-Nrf2/BDNF pathway. Both 15 g/kg and 30 g/kg doses upregulated Nrf2, kelch-like ech-associated protein 1 (Keap1), heme oxygenase-1 (HO-1), NAD(P)H quinone oxidoreductase 1 (NQO1), and glutathione peroxidase 3 messenger RNA (GPX3mRNA) levels in the cortex, with 30 g/kg exerting stronger effects on Keap1. XYS also increased p-Akt/Akt ratios and elevated TrkB protein expression [67]. Similarly, Ji et al. demonstrated that XYW at 0.93 and 1.86 g/kg alleviated depression-like behaviors in OB rats by enhancing the expression of Nuclear factor erythroid 2-related factor 2(NFE2L2), KEAP1, glutathione peroxidase 3(GPX3), heme oxygenase 1(HMOX1), superoxide dismutase 1(SOD1), NAD(P)H dehydrogenase quinone 1(NQO1), and BDNF, while reducing ROS levels, implicating the PIK3CA-AKT1-NFE2L2/BDNF axis in oxidative stress modulation and neuroprotection [68].

*Regulation of metabolism* In a model of chronic immobilization stress (CIS), Pan et al. showed that CIS-induced depressive behavior was associated with reduced appetite, insulin, and lipid levels. XYS reversed these effects, restoring SHIP2 expression while reducing Akt, p-Akt, and p85 expression in the liver, suggesting a mechanism involving hepatic PI3K/Akt activation via SHIP2 modulation [69]. Another study demonstrated that XYS alleviated CUMS-induced depressive symptoms and hyperglycemia by modulating the Leptin Receptor (LepR)-Signal Transducer and Activator of Transcription 3 (STAT3)/PI3K cascade. XYS downregulated leptin and STAT3 expression, reduced low-density lipoprotein (LDL) levels, and normalized the expression of appetite-regulating genes such as Proopiomelanocortin (POMC), Agouti-Related Protein (AGRP), and Neuropeptide Y (NPY) mRNA, indicating its role in both metabolic and mood regulation [70]

*Anti-inflammatory* Lipopolysaccharide (LPS)-induced depression models demonstrated that XYW restored 5-HT levels and reduced indoleamine 2,3-dioxygenase (IDO) expression by activating the NGF/BDNF-TrkA/TrkB-CREB signaling pathway, thereby alleviating neuroinflammation and promoting neuronal survival [71].

#### XYS active compounds regulate the PI3K/Akt signaling pathway to alleviate depression

***Saikosaponin (SS),*** with a molecular formula of C₄₂H₆₈O₁₃, is a natural triterpenoid saponin primarily extracted from *Bupleuri Radix* and other traditional Chinese medicinal herbs. These compounds exhibit a broad range of biological activities [72], including anti-inflammatory, antioxidant, and neuroprotective effects, as well as the ability to modulate neurotransmitter systems and maintain neuronal integrity and signal transduction [73]. Among them, *Saikosaponin A* (SSa), *Saikosaponin B* (SSb), and *Saikosaponin D* (SSd) have been identified as the main active constituents with antidepressant properties through the modulation of various signaling pathways[74]. One study demonstrated that 5 mg/kg SSa significantly alleviated depression-like behaviors in MCAO and CUMS rats by regulating the p-CREB/BDNF signaling pathway and inhibiting hippocampal neuronal apoptosis [75]. Additionally, SSd was shown to downregulate the expression of phosphorylated Akt, Foxg1, and fibroblast growth factor 2 (FGF2) in the hippocampus, implicating modulation of the Akt/Foxg1 pathway [76]. Sun conducted two experiments confirming the antidepressant effects of total saikosaponins (TSS). In the first, TSS (25 and 50 mg/kg) significantly reduced immobility time in the forced swim test (FST) in ICR mice, with 25 mg/kg showing the strongest effect. In the second experiment, 25 mg/kg TSS alleviated depression-like behaviors in chronic corticosterone-treated mice and enhanced expression of synaptic proteins. Moreover, TSS increased phosphorylation of GluR1 at Ser845 (an AMPA receptor subunit), ERK, Akt, and mTOR in the hippocampus, suggesting involvement of AMPA receptor and mTOR pathway regulation[77].

***Paeoniflorin (PA; C₂₃H₂₈O₁₁)***, mainly isolated from *Radix Paeoniae Alba*, possesses neuroprotective, anti-inflammatory, analgesic, and anti-arrhythmic properties [78]. Numerous studies have shown that PA exerts antidepressant effects by elevating monoamine neurotransmitter levels, modulating the HPA axis, promoting neurogenesis, and enhancing neuroprotection [79]. Liu et al. reported that PA downregulated glutamate levels in the CA1 region and upregulated PSD-95 and SYP expression by modulating Homer1-mGluR5 and mTOR pathways [80]. Similarly, Chen found that PA alleviated postpartum depression-like behaviors in rats, increased serum estradiol and progesterone levels, and activated the TSPO and BDNF/mTOR pathways, thereby reducing inflammatory cytokine release [81].

***Ferulic acid (FA; C₁₀H₁₀O₄)***, chemically known as 3-methoxy-4-hydroxycinnamic acid, is derived from herbs such as *Angelica Sinensis* and *Radix Paeoniae Alba* [82]. FA exhibits antidepressant activity through modulation of monoamine neurotransmitters, inhibition of neuroinflammation, and attenuation of oxidative stress [83]. One study demonstrated that FA reduced LPS-induced depression-like behaviors in mice by regulating IDO, BDNF, and inflammation-related factors [84]. Additionally, Li et al. found that SIRT6 in the hippocampus contributed to depressive phenotypes by inhibiting the AKT/CRMP2 pathway. At the same time, FA treatment suppressed SIRT6 activity and restored AKT/CRMP2 signaling, thereby improving depressive behaviors in CUS rats [85].

Beyond individual active compounds, both the decoction and volatile oil components of XYS exhibit therapeutic effects. Xie et al. found that volatile oils from a disassembled XYS formula—composed of Chaihu (*Bupleuri Radix*), Danggui (*Angelicae Sinensis Radix*), and Bohe (*Menthae Haplocalycis Herba*) (CDB)—improved LPS-induced depression-like behaviors, enhanced Nissl body synthesis in the CA3 region, and conferred neuroprotection. These effects were linked to the activation of PI3K/Akt and Nrf2/HO-1 pathways, as well as increases in glutathione (GSH) and superoxide dismutase (SOD) activity [86]. Furthermore, the ethyl acetate fraction of XYS (XYSEF) alleviated depressive behaviors in LPS-induced mice, potentially through the promotion of hippocampal neurogenesis, inhibition of apoptosis, and suppression of the IGF-1Rβ/PI3K/Akt signaling axis [87].

In summary, XYS and its bioactive constituents ameliorate depression-like behaviors in animal models by targeting the PI3K/Akt signaling pathway. Their therapeutic mechanisms include inhibition of hippocampal neuronal apoptosis, suppression of neuroinflammation, modulation of liver metabolism, improvement of glucose tolerance, reduction of synaptic loss, mitigation of oxidative stress, attenuation of glutamate excitotoxicity, neuroprotection, and promotion of myelin regeneration and structural integrity within neural circuits.

### NLRP3 signaling pathway

The NLRP3 inflammasome is a multiprotein intracellular complex belonging to the NOD-like receptor (NLR) family, primarily composed of the NLRP3 receptor protein, the adaptor apoptosis-associated speck-like protein (ASC), and the effector protease caspase-1 [88]. Under physiological conditions, NLRP3 remains in an inactive conformation, often forming a bicircular cage-like structure, and is primarily localized to the membranes of astrocytes and microglia [89]. However, activation of toll-like receptors (TLRs), NF-κB signaling, reactive oxygen species (ROS) generation, and Ca^2^⁺ flux can trigger the assembly of the NLRP3 inflammasome [90].

Upon stimulation, NLRP3 recruits ASC via PYD-PYD domain interactions. ASC then binds pro-caspase-1 through CARD-CARD interactions, initiating the assembly of the functional inflammasome complex [91]. Activated caspase-1 subsequently cleaves pro-IL-1β and pro-IL-18 into their active forms, IL-1β and IL-18, triggering a broad inflammatory response [91]. Furthermore, caspase-1 cleaves gasdermin D (GSDMD), which forms membrane pores that lead to pyroptotic cell death and the release of inflammatory mediators. This amplifies neuroinflammation, disrupts neuronal function, and contributes to the development and progression of depression[92–94].

Elevated protein and mRNA expression of NLRP3 and ASC have been reported in postmortem brain tissue from patients with depression [95]. Animal studies similarly show that NLRP3 expression and proinflammatory cytokines such as IL-1β and IL-6 are significantly increased in the brains of CUMS-induced depressive rats. Pharmacological inhibition or genetic knockdown of NLRP3 in these animals significantly alleviated or reversed depressive-like behaviors[96, 97]. Notably, several studies have shown that XYS and its active components exert antidepressant effects through modulating the NLRP3 signaling pathway.

#### XYS regulates NLRP3-related signaling to ameliorate depression

*Neuroprotection* Chen et al. explored the underlying mechanisms and found that XYS suppressed NLRP3, caspase-1, and IL-1β protein levels in the cerebral cortex of CIS rats. It also downregulated endoplasmic reticulum (ER) stress- and apoptosis-related mRNAs, including GRP78, CHOP, and caspase-12, indicating that XYS may alleviate depression by modulating the NLRP3–ER stress–apoptosis axis [98].

*Anti-inflammatory* XYS has been shown to suppress activation of the TLR4/NLRP3 axis, thereby reducing NF-κB and IL-1β levels in CUMS rats. It also enhances the integrity of the gut and blood–brain barriers by upregulating tight junction proteins, such as claudin-1 and zonula occludens-1 (ZO-1), suggesting a role for the microbiota–TLR4/NLRP3–barrier axis in its antidepressant effect [99]. In mice subjected to chronic immobilization stress (CIS) under isolated housing conditions, XYS downregulated the expression of TLR4, MyD88, NF-κB-p65, TAK1, IRAK1, TRAF6, NLRP3, ASC, and caspase-1. Correspondingly, it reduced IL-6, IL-1β, and TNF-α levels in both colon tissue and serum, thereby mitigating colonic inflammation [100].

#### Active compounds of XYS modulate NLRP3 signaling to alleviate depression

Cheng et al. examined the effect of PA, an active compound of XYS, in LPS-induced depressive mice. PA suppressed pro-inflammatory cytokine production and microglial activation by inhibiting the TLR4/NF-κB/NLRP3 pathway. It also elevated FGF-2 levels and dendritic spine density in neurons. Notably, co-administration of the FGFR1 inhibitor SU5402 abolished these effects, indicating that PA may exert neuroprotective and antidepressant actions through activation of the FGF-2/FGFR1 pathway [101].

*P. Cocos water extract* (PCW), derived from the sclerotia of *Poria cocos* [102], has been found to exert immunomodulatory, antitumor, and antioxidant effects [103]. In CUMS rats, PCW significantly improved depressive behaviors, enhanced hippocampal neurogenesis, and restored neurotransmitter levels, including BDNF, 5-HT, 5-HIAA, DA, and NE. It also reduced pro-inflammatory markers, such as IL-1β, IL-8, TNF-α, and NLRP3, in a dose-dependent manner [104].

In a separate study, Liu demonstrated that FA reduced TNF-α and NF-κB levels and inhibited NLRP3 inflammasome activation in CUMS mice, further supporting the role of FA in suppressing inflammation-associated depressive phenotypes via the NF-κB/NLRP3 axis[105].

In summary, XYS and its active constituents modulate NLRP3-related signaling pathways, reduce neuroinflammation, attenuate ER stress and apoptosis in the cerebral cortex, and inhibit microglial activation. These effects collectively contribute to their efficacy in ameliorating depressive-like behaviors in animal models.

### NF-κB signaling pathway

NF-κB was initially identified in B lymphocytes due to its ability to bind the κB enhancer sequence of immunoglobulin light chains. It comprises five subunits—p65 (RelA), RelB, c-Rel, p50, and p52—which interact through their N-terminal Rel homology domains (RHD) to form homo- or heterodimers [106]. NF-κB is primarily activated through two distinct pathways: the classical and the non-classical.

The classical pathway is initiated by members of the TNF receptor superfamily, which recruit adaptor proteins such as TNF receptor-associated death domain (TRADD), leading to activation of the IκB kinase (IKK) complex. IKK phosphorylates the inhibitor IκB, marking it for ubiquitination and degradation, thereby liberating NF-κB dimers to translocate into the nucleus. Once in the nucleus, NF-κB induces transcription of target genes involved in inflammation, immunity, cell proliferation, and survival [107].

In the non-classical pathway, receptors such as lymphotoxin-β receptor (LTβR) and CD40 trigger NF-κB-inducing kinase (NIK), which activates IKKα. IKKα phosphorylates p100, leading to its processing into p52 and the release of the RelB/p52 dimer, which then enters the nucleus to regulate the expression of target genes. This pathway is critical for lymphoid organ development, B-cell maturation and survival, and the immune response[108].

Both NF-κB pathways are involved in the pathophysiology of depression, particularly through their role in neuroinflammation and central nervous system (CNS) dysfunction [109]. Chronic activation of NF-κB promotes the release of inflammatory cytokines [110], leading to amplified immune responses, neurotransmitter imbalances, impaired neuroplasticity, increased oxidative stress [111], and dysregulation of the HPA axis [112]. Elevated levels of IL-6 in depressive rat models have been significantly reduced following treatment with NF-κB antagonists, resulting in improved behavioral outcomes [113]. Inhibition of NF-κB activity also reduces microglial activation, preserving CNS homeostasis and supporting antidepressant effects [114]. Moreover, NF-κB signaling contributes to neuronal apoptosis via transcriptional upregulation of pro-apoptotic genes such as *P53* and *c-Myc*, exacerbating neural structural and functional impairments associated with depression[115].

Experimental studies have demonstrated that XYS and its active constituents modulate NF-κB-related pathways, significantly alleviating depressive-like behaviors in animal models.

#### XYS regulates NF-κB signaling to alleviate depression

XYS has been shown to mitigate inflammation in CUMS-induced depressive mice by targeting the MYDGF/MAP4K4/NF-κB signaling axis. It suppressed IL-6 and TNF-α expression in the brain and inhibited the expression of MYDGF, PKC, MAP4K4, p-p65, p65, p-IκBα, and IκBα, as confirmed by molecular docking studies [116].

Zhu et al. further demonstrated that XYS mimics the effect of A2AR antagonists by downregulating the expression of p-ERK, NF-κB, and A2AR in CIS rats while enhancing Na⁺/K⁺-ATPase activity and ATP levels. XYS also improved synaptic integrity, promoted axonal growth, and reduced microglial activation. Behavioral tests indicated a significant improvement in anxiety-like behaviors in chronic restraint stress (CRS) rats, suggesting that XYS exerts antidepressant effects via modulation of the A2AR-ERK-NF-κB pathway [117].

#### Active components of XYS modulate NF-κB signaling in depression

Wang et al. reported that SSB2 ameliorates microglial activation and depressive symptoms by inhibiting neuroinflammation and endoplasmic reticulum stress through the TLR4/NF-κB pathway [118]. Su et al. found that SSd alleviates LPS-induced depressive-like behaviors by regulating the HMGB1/TLR4/NF-κB axis. ELISA analysis revealed decreased levels of IL-1β, IL-6, and TNF-α in both brain tissue and serum following SSd treatment. Immunohistochemistry and Western blot assays showed that SSd inhibited HMGB1 nuclear translocation and reduced TLR4 expression in LPS-treated mice [119].

Wang also demonstrated that PA alleviates depressive-like symptoms in systemic lupus erythematosus (SLE)-induced MRL/lpr mice by suppressing HMGB1/TLR4/NF-κB signaling. PA restored behavioral parameters such as sucrose preference and immobility time in tail suspension and forced swim tests. It also reduced levels of IL-6, IL-1β, and TNF-α in both serum and hippocampus, thereby mitigating neuroinflammation [120]. Additionally, a recent study revealed that PA downregulated gasdermin D (GSDMD) expression in hippocampal microglia of reserpine (RESP)-induced mice, while enhancing CASP-11, CASP-1, NLRP3, and IL-1β expression. Co-treatment with VX-765, a CASP-1 inhibitor, further decreased inflammasome-related protein levels and enhanced PA’s antidepressant effects, indicating that PA may alleviate depression by inhibiting CASP-11-dependent pyroptosis [121].

Bai et al. showed that PA regulates the TLR4/NF-κB pathway and reduces hippocampal inflammation, improving depression associated with neuropathic pain [122]. In another study, Huang et al. employed a UCMS rat model to evaluate the antidepressant effects of *Poria cocos* water extract (PCW). After 28 days of treatment, PCW significantly reduced levels of p38, NF-κB, and TNF-α in the cortex, while normalizing 5-HT turnover [104]. Zhang further found that PCW attenuated anxiety-like behaviors in chronic sleep-deprived (CSD) rats by suppressing TNF-α/NF-κB signaling. Multi-omics analyses indicated that PCW also modulated gut microbiota and metabolic profiles, contributing to its antidepressant efficacy [123].

*Isoliquritin*, a flavonoid derived from licorice roots, exhibits anti-inflammatory, antioxidant [124], and antiviral activities [125]. Li’s study demonstrated that 30 mg/kg isoliquiritin reduced neuronal apoptosis and microglial activation in the hippocampus of LPS-induced depressive mice by regulating the miRNA-27a/SYK/NF-κB axis. Isoliquiritin downregulates p-NF-κB, NLRP3, and SYK at both mRNA and protein levels. Importantly, its antidepressant effect was abolished by miRNA-27a inhibition, underscoring the pathway's regulatory significance[126].

In summary, XYS and its active components modulate the NF-κB signaling pathway, inhibit microglial activation, restore synaptic structure, and reduce inflammation, thereby effectively alleviating depressive-like symptoms in animal models.

### MAPK signaling pathway

The mitogen-activated protein kinase (MAPK) pathway is a critical intracellular signaling cascade that involves serine/threonine kinases, which transduce extracellular signals to intracellular targets. It is activated by various stimuli, such as growth factors, glutamate, hormones, cellular stress, and pathogens, and governs essential cellular processes, including proliferation, differentiation, apoptosis, and inflammation [127]. The core MAPK cascade comprises three kinase tiers: MAPK kinase kinase (MAPKKK), MAPK kinase (MAPKK), and MAPK. The MAPK family includes ERK1/2, ERK5, c-Jun N-terminal kinase (JNK), and p38 MAPK, all of which are activated through sequential phosphorylation, typically culminating in MAPK activation by MAPKK [128].

Evidence suggests that aberrant activation of MAPK signaling is implicated in depression. Activation of p38 MAPK, JNK, and ERK can induce pro-inflammatory cytokine production, disrupt synaptic plasticity, impair memory, and alter stress responses [129]. Among these, the JNK and ERK1/2 pathways are particularly relevant to depression. JNK, a 46/54 kDa kinase with three isoforms (JNK1, JNK2, JNK3), exhibits differential tissue expression: JNK1 and JNK2 are ubiquitous, while JNK3 is predominantly expressed in the CNS [130]. J Inhibition of JNK in neonatal hippocampal granule cells mitigates depressive-like behavior, and brain-wide JNK1 inhibition enhances neurogenesis [131]. Moreover, JNK activation promotes inflammation and microglial activation [132]. Mice lacking JNK1 or treated with JNK inhibitors show increased hippocampal neurogenesis and attenuated depressive phenotypes [133]. Several studies demonstrate that XYS and its active constituents can suppress MAPK pathway activation, contributing to antidepressant effects.

#### XYS regulates MAPK-related signaling to alleviate depression

Compared to fluoxetine-treated controls, XYS significantly downregulated JNK, phospho-JNK (p-JNK), and phospho-c-Jun (p–c-Jun) in the hippocampus, leading to improved anxiety-like behavior in CIS rats. These findings suggest that XYS mitigates depressive symptoms by modulating the hippocampal JNK pathway [134]. Similarly, Yang et al. reported that XYS alleviated LPS-induced depression-like behavior in mice by suppressing neuroinflammation and the JNK pathway. Following XYS treatment, mice exhibited increased open-field and elevated plus-maze exploration times. Biochemical analyses revealed increased norepinephrine and adrenaline levels, alongside reduced mRNA expression of IL-1β, JNK3, c-Jun, and c-Fos, as well as decreased protein levels of Iba-1, c-Fos, JNK, and p-JNK in the hippocampus [135]

#### Active components of XYS modulate MAPK signaling to improve depression

In CUMS rats, treatment with paeoniflorin (PA) restored sucrose preference and enhanced locomotor activity. PA also attenuated hippocampal neuronal damage by modulating the BDNF-ERK-CREB pathway. However, co-treatment with U0126, a selective ERK inhibitor, abolished these effects, confirming PA's dependence on the ERK-CREB axis [136]. Further studies confirmed that PA significantly alleviates depression-like behavior in chronic restraint stress (CRS) mice through ERK1/2 pathway regulation [137].

*Oleanolic acid* (OA), a triterpenoid compound (C₃₀H₄₈O₃) found in *Bupleuri Radix*, *Radix Paeoniae Alba*, and licorice, exhibits anti-inflammatory, antioxidant, antiviral, and metabolic regulatory effects [138]. Yi et al. demonstrated that OA activates the BDNF-ERK-CREB signaling pathway in the hippocampus, exerting antidepressant effects. Specifically, OA upregulates miR-132 and promotes hippocampal neurogenesis in CUMS mice, thereby alleviating anxiety-like behaviors [139].

In addition, in vitro studies revealed that chlorogenic acid (CF) inhibits the NMDAR-CaMKII-MAPK signaling axis, suppresses oxidative stress, and mitigates mitochondrial apoptosis in PC12 cells. Behavioral assays demonstrated that CF significantly reduced immobility time in both the tail suspension test (TST) and the forced swim test (FST), supporting its potential antidepressant properties [140].

In summary, XYS and its active components exert antidepressant effects by modulating MAPK-related pathways. These interventions reduce neuronal apoptosis, restore hippocampal structure and function, and inhibit neuroinflammatory processes, thereby alleviating depressive behaviors in animal models.

### Other signaling pathways

Beyond the well-characterized mechanisms, XYS and its active components also exert antidepressant effects through additional pathways, including blood-oxygen-level-dependent functional magnetic resonance imaging (BOLD-fMRI) signals, receptor for advanced glycation end-products (RAGE)-mediated inflammation, and the microRNA-200 (miR-200)/nuclear receptor subfamily 3 group C member 1 (NR3C1) axis.

#### XYS regulates other signaling pathways to alleviate depression

*Anti-inflammatory* Yan et al. demonstrated that XYS, comparable to FLX, significantly attenuated depressive-like behavior in CUMS mice. Specifically, XYS suppressed RAGE mRNA and protein expression in the cingulate gyrus while enhancing its functional connectivity, potentially reducing RAGE-mediated inflammatory signaling and preserving neural network integrity [141].

Using network pharmacology, a study identified 23 depression- and necroptosis-related targets modulated by XYS. Experimental validation in CUMS mice confirmed that XYS downregulated RIPK1, RIPK3, MLKL, and p-MLKL expression in the hippocampus. Moreover, XYS increased IL-1β expression and decreased Lipocalin-2 levels, suggesting that it inhibits necroptosis and neuroinflammation through modulation of the RIPK1-RIPK3-MLKL signaling axis [142]

Li et al. investigated whether XYS exerts anxiolytic effects via the hippocampal TNF-α/JAK2-STAT3 pathway. In CIS rats, XYS treatment, comparable to the JAK2 inhibitor AG490, alleviated anxiety-like behavior, reduced hippocampal damage, and inhibited activation of the TNF-α/JAK2-STAT3 pathway. However, it did not significantly affect apoptosis-related markers [143]. Further evidence shows that XYS can ameliorate cerebral cortical inflammation and pathology in CIS rats, primarily by activating the cAMP signaling pathway. XYS increased cAMP and CREB1 mRNA expression in the cortex and reduced serum levels of PGE2 and SP [144].

*Neuroprotection* In single-housed CUMS mice, XYS significantly increased the expression of caspase-3 and Bax in the prefrontal cortex, while elevating Bcl-2 and NR3C1 levels in primary cortical neurons, thereby reducing neuronal apoptosis. These findings suggest that XYS may mediate antidepressant effects by upregulating the miR-200/NR3C1 signaling pathway [145]. Jiang et al. further showed that XYS alleviated depressive symptoms in CIS rats and increased BDNF levels in vivo, potentially via modulation of the CRF1R pathway in the amygdala [146].

*Regulation of metabolism* Ma et al. proposed that XYS exerts antidepressant and anti-anorexia effects via the NES1-OT-POMC neural circuit. In CIS rats, XYS treatment reversed elevated NES1 levels in the serum and paraventricular nucleus, and downregulated hypothalamic POMC, OT, and MC4R expression [147]. Additionally, XYS modulated the leptin–Ob-R–POMC pathway to restore food intake and body weight. In CIS rats, leptin normally activates Ob-R in the arcuate nucleus, leading to increased α-MSH expression and suppressed appetite. XYS counteracted this process by modulating the leptin signaling axis [148]. Tang et al. reported that XYS ameliorated both depressive-like behavior and glucose intolerance in CSDS mice by upregulating serum adiponectin and activating the AdipoR1/AMPK/ACC signaling pathway [149].

#### Active components of XYS regulate other signaling pathways to alleviate depression

Tong et al. found that saikosaponin A (SSa) markedly relieved depressive symptoms in CSDS mice by enhancing hippocampal neurodevelopment and dendritic spine density. SSa also upregulated Ten-Eleven Translocation 1 (Tet1), Notch, Delta-Like 3 (DLL3), and BDNF expression. Knockdown of Tet1 attenuated these effects, suggesting that SSa mediates its antidepressant effects through the Tet1/DLL3/Notch signaling pathway [150].

Xu et al. showed that lysophosphatidic acid receptor 1 (LPA1)-mediated neuronal apoptosis plays a key role in LPS-induced depression. SSd significantly reduced RhoA and ROCK expression in SH-SY5Y cells and decreased LPA1 expression and apoptosis in BV2 microglia, indicating that SSd improves depressive symptoms by inhibiting the LPA1/RhoA/ROCK2 signaling cascade [151].

*Albiflorin* (AF; C₂₃H₂₈O₁₁), a monoterpenoid glycoside derived from Paeonia species, exhibits a wide range of pharmacological activities, including anti-inflammatory, hepatoprotective, cardiovascular, and neuroprotective effects [152]. AF also possesses antidepressant properties [153]. In CRS rats, AF mitigated the adverse effects of sustained glucocorticoid exposure, increased levels of 5-HT, 5-HIAA, NE, DA, and BDNF, and elevated hippocampal protein expression. AF treatment also reduced NO and cGMP concentrations and downregulated 5-HT2AR mRNA and protein expression, suggesting that its antidepressant effects may be mediated via NO-dependent hippocampal signaling pathways [154].

Yang et al. first identified the antidepressant effect of DL-3-n-butylphthalide (NBP). In LPS-induced depressed rats, NBP attenuated depressive-like behavior, suppressed the expression of IL-1β and IL-6, downregulated the NF-κB pathway to reduce inflammation, and activated Nrf2 signaling to counteract oxidative stress [155].

**Fig. 4:**
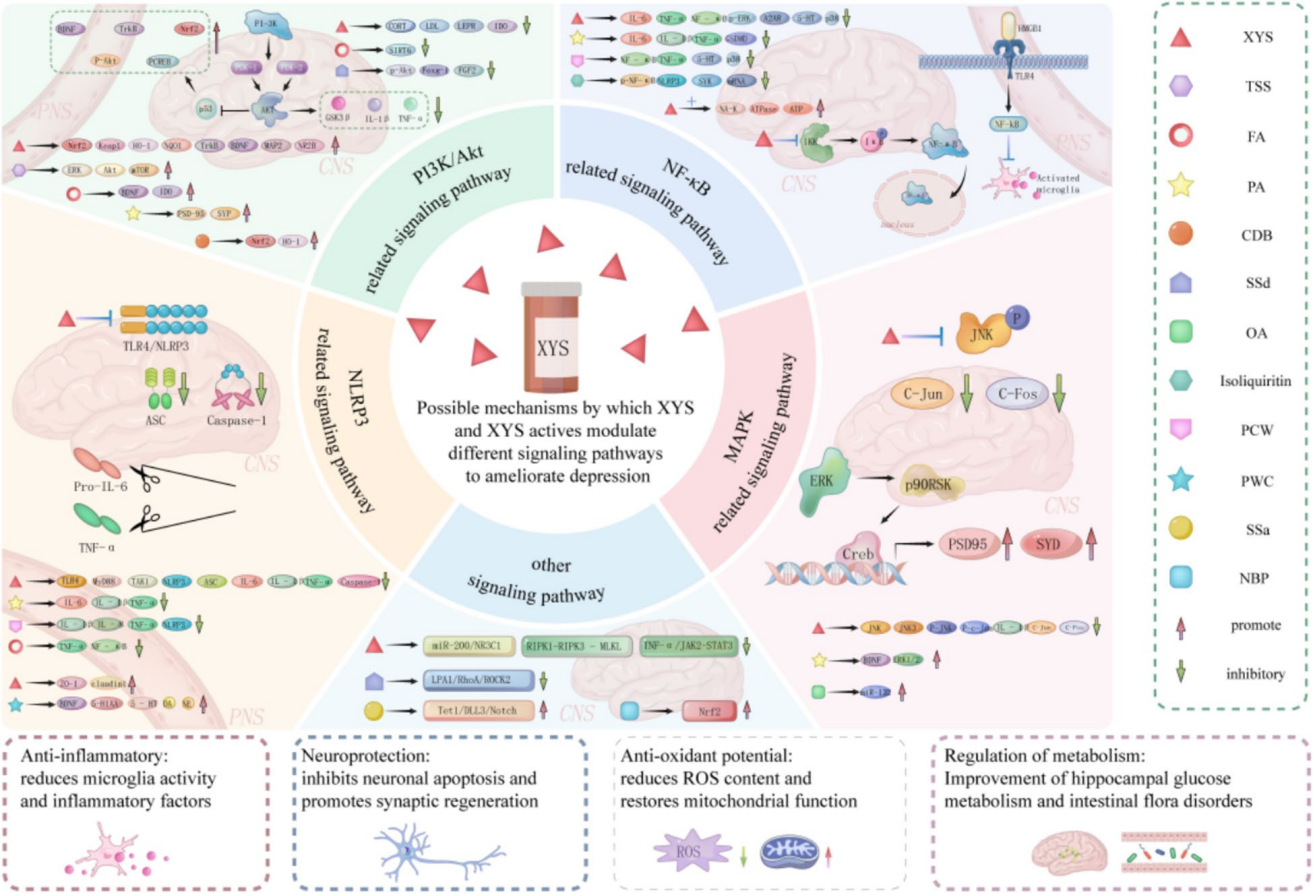
Possible mechanism of XYS and XYS active substances regulating different signaling pathways to improve depression

Possible mechanisms by which XYS and XYS active substances modulate different signalling pathways to ameliorate depression are shown in Fig. 4, and the mechanisms by which XYS and XYS-active substances modulate different signalling pathways in antidepressants are shown in Table 2.

**Table 2:** Effect of XYS and XYS-active substances on depression through modulating different signalling pathways

Model type	Experimental Intervention	Control Intervention	Period	Effect indicators	mechanism	Signaling Pathway	References
CUMS male SD rat	XYS2.224 g/kg/day	FLU5.4 mg/kg/day	4 weeks	↑MAP2,PSD-95, SYN,BDNF, trkB, p-trkB,PI3K,Akt,p-Akt	Reduce hippocampal synaptic loss and alleviate hippocampal pathological damage	PI3K/Akt and BDNF/trkB	[48]
CUMS male SD rats	XYW5,10,20 g/kg/day	RU486 25 mg/kg	/	↑GR,p-GR,BDNF,Cx43; ↓CORT,c-Src	Ameliorate hippocampal neuron damage	Cx43/GR/BDNF	[64]
CUMS male SD rats	XYS2.224 g/kg/day	FLU2mg/kg/day	2 weeks	↑Glutamate,CORT,MAP2,NR2B,PI3K	Improve hippocampal neuron apoptosis	NR2B and PI3K/Akt	[65]
VaD-CRS male C57BL/6 J mice	XYS5-20 g/kg/day	FLU10mg/kg/day	4 weeks	↑MBP,MAG,MOG,p-PI3K/PI3K,p-AKT/AKT,p-mTOR/mTOR	Promote myelin regeneration in neural circuits and increase myelin structural integrity	PI3K / AKT / Mtor	[66]
OB male SD rats	XYS15or30g/kg/day	FLU0.01 g/kg/day	30 days	↑Nrf2,Keap1,HO-1,NQO1,GPX3mRNA,OGG1mRNA,SOD1,HO -1,BDNF	Improve oxidative stress	PI3K-AKT-Nrf2 / BDNF	[67]
OB male SD rats	XYW0.93and1.86 g/kg/day	FLU0.01 g/kg/day	30 days	↑NFE2L2、KEAP1、GPX3、HMOX1、SOD1、NQO1、BDNF mRNA; ↓ROS	Alleviation of oxidative stress and the enhancement of neuroprotective effects	PIK3CA-AKT1-NFE2L2/BDNF	[68]
CIS male SD rats	XYS1.845 g/kg/day	Rosiglitazone3mg/kg/day	21 days	↑CHOL,HDL-C,LDL-C,TG; ↓SHIP2,Akt,Mrna,p-AKT	Liver metabolism	PI3K/Akt	[69]
CUMS male SD rats	XYS2.224 g/kg/day	FLU2mg/kg/day	6 weeks	↑AGRP,NPY; ↓LDL,LEPR,STAT3,POMC	Improve glucose tolerance	LepR-STAT3/PI3K	[70]
LPS male SD rats	XYW1.86 g/kg/day	sterile saline1.86 g/kg/day	14 days	↑BDNF、NGF、TrkB、TrkA、CREB、p-CREB、p-CREB/CREB、SYP; ↓IL-6、TNF-α、IDO	Inhibited of neuronal inflammation and promoted the recovery of nerve injury	NGF/BDNF-TRKA/TrkB-CREB	[71]
MCAO + CUMS male SD rats	SSa5mg/kg/d	FLU5mg/kg/d	24 days	↑p-CREB、BDNF、Bcl-2; ↓Bax、Caspase-3	Inhibit neuronal apoptosis	p-CREB/BDNF	[75]
PC12 cells	SSd4mg,8/kg/d	30 mg/kgLY294002	7 days	↓p-Akt,Foxg-1,FGF2	Improve hippocampal cognition	Akt/Foxg-1	[76]
CORT male C57BL/6 J mice	TSS25mg/kg/d	citalopram10mg/kg	14 days	↑GluR1 Ser845,p-Akt, p-ERK,p-Mtor,synapsin-1,PSD-95	Expression of synaptic proteins	mTOR	[77]
PPD female SD rats	PA20mg/kg	FLU3.0 mg/kg	2 weeks	↑E2,Allo,Erα,Erβ; ↓Cor,IL-1β,ERα/ERβ	Hormone levels, inflammatory factors	TSPO and BNDF-Mtor	[81]
CUS male C57BL/6 mice	FA40,80 mg/kg/d	/	14 days	↑H3K56ac,H3K9ac,AKT; ↓SIRT6,CRMP2	Weakening hippocampal neuron damage	SIRT6/AKT/CRMP2	[85]
LPS male C57BL/6 mice	CDB36.8,73.6 mg/kg/d	FLU10mg/kg/d	13-15 days	↑Iba-1,BDNF,p-PI3K/PI3K,p-Akt/Akt,Nrf2,HO-1,SOD1; ↓p-p65/p65,IL-1β,Iba-	Oxidation level, reducing inflammation	PI3K/Akt/Nrf2	[87]
CIS male SD rats	XYS2.224 g/kg/day	FLU2mg/kg/day	21 days	↓mRNA,NLRP3,Caspase-1,IL-1β,GRP78,CHOP	Inhibiting endoplasmic reticulum stress and apoptosis molecules in the cerebral cortex	NLRP3	[98]
CUMS male SD rats	XYS23g/kg/day	PX, 2.1 mg/kg	/	↑claudin 1 and ZO-1; ↓Bacteroidetes/Firmicutes ratio, the abundances of Bacteroides, and Corynebacterium,	Maintained the integrity of gut and blood–brain barriers	microbiota-TLR4/NLRP3	[99]
CIS male SD rats	XYS2.224 g/kg/day	FLU2mg/kg/day	7 days	↓IL-1β,IL-6,TNF-α,TLR4, MYD88, NF-κB-p65, TAK1,TRAF6,ASC, and caspase-1	Reduce inflammatory factors	TLR4/NLRP3	[100]
LPS male ICR mice	PA20,40,80 mg/kg/d	FLU20mg/kg/d	7 days	↑FGF-2; ↓IL-1β,IL-6,TNF-α,Iba1,COX-2	Inhibit the activation of microglia, reduce the release of inflammatory factors, and minimize neuronal damage	FGF-2/FGFR1	[101]
UCMS male SD rats	PCW100,300,900 mg/kg/d	FLU20mg/kg/d	28 days	↓p38,NF-κB,TNF-α	Regulating monoamine neurotransmission and inactivating inflammation	NF-κB	[104]
CUMS male ICR mice	FA20,40,80 mg/kg/d	FLU20mg/kg/d	28 days	↓IL-1β,IL-6,TNF-α,NF-κB,NLRP3,caspase-1	Anti-inflammatory	NF-κB	[105]
CUMS male C57BL/6 mice	XYSgranules 1.258 g/kg/d	FLU1.3 g/kg/d	12 weeks	↓IL-6,TNF-α,CORT, hs-CRP, NFκB, FIB, Lp-PLA2,MYDGF, PKC, MAP4K4, P-p65, p65	Reduce inflammation	MYDGF/MAP4K4/NF-κB	[116]
CRS male SD rats	XYS2.224 g/kg/day	SCH 58261 0.05 mg/kg/d	21 days	↑NA–K ATPase,ATP; ↓p-ERK,NF-κB,A2AR	Inhibit microglial activation and improve synapses	A2AR-ERK-NF-κB	[117]
CUMS male ICR mice	SSb2	/	7 days	↑GSH,SLC7A11,FTH,GPX4,Nrf2; ↓ACSL4,TFR1	Reduce central nervous system inflammation and improve hippocampal nerve damage	TLR4/NF-κB	[118]
LPS male ICR mice	SSd1mg/kg/d	sterile saline	7 days	↓TLR4, p-IκB-α, NF-κBp65,IL-1β,IL-6,TNF-α	Inhibit microglial activation and neuroinflammation	HMGB1/TLR4/NF-κB	[119]
male MRL/lpr mice	PA20mg/kg/d	/	/	↓TNF-α,IL-6,IL-1β,HMGB1,TLR4,MyD88,P-NF-κBp65,p-IκBα	Reduce inflammation	HMGB1/TLR4/NF-κB	[120]
RESP male C57BL/6 mice	PA10,20,40 mg/kg/d	FLU20mg/kg/d	4 days	↓caspase(CASP) 11,CASP-1,NLRP3,IL-1β	Activation of microglia and neuroinflammation	CASP-11/GSDMD	[121]
NP male Balb/c mice	PA50,101 mg/kg	sterile saline	14 days	↓TNF-α IL-1β, and IL-6,Iba-1,TLR4, MyD89	Reduce pathological damage to hippocampal cells, lower levels of pro-inflammatory cytokines, and inhibit overactivation of microglia	TLR4/NF-kB	[122]
CSD male Wistar rats	PCW9.375,18.75,37.5 g/kg/d	estazolam0.18 mg/kg	21 days	↑BHMT,LS-L-TA,L-TA,p-Ikkα/β; ↓BA-CoA,TNF-α,IL-6,IL-1β	Regulating intestinal flora, regulating metabolic disorders, and inhibiting inflammatory pathways	TNF-α/NF-κB	[123]
LPS male C57BL/6 J mice	Isoliquiritin 30 mg/kg/d	FLU20mg/kg/d	14 days	↑miRNA-27a; ↓p-NF-κB, NLRP3,Caspase-1, IL-1β,GSDMD-N	Neuronal apoptosis inhibits activation of microglia cells	miRNA-27a/SYK/NF-κB	[126]
CIS male SD rats	XYS3.9 g/kg/d	Deionized water10 mL/kg/d	13 days	↓P-JNK,JNK,P–c-Jun,Cyt-C	Hippocampal nerve	JNK	[134]
LPS male C57BL/6 J mice	XYS6.012 5,12.025,24.050 g/kg	FLU2.6 mg/kg	14 days	↑NE;↓IL-1β,Iba-1,c-Fos,c-Jun,JNK、p-JNK	Inhibit neuroinflammation	JNK	[135]
CUMS male SD rats	PA30-60 mg/kg/d	U0126 1µL/min/d	5 weeks	↑ERK1,ERK2,CREB,ERK,p-ERK	Reduce hippocampal damage	ERK-CREB	[136]
CRS male C57BL/6 J mice	PA10,30,60 mg/kg	U0126 1µL/min/d	5 weeks	↑p-ERK1,p-ERK2,CREB,BDNF	Reduce chronic stress	ERK1/2	[137]
CUMS male ICR mice	OA20mg/kg/d	FLU20mg/kg/d	3 weeks	miR-132,BDNF,PSD 95	Neurotrophic actions	BDNF–ERK–CREB	[139]
PC12 cells	CF0.2, 2,20 μmol/L	/	/	↓p-NR2B, p-CaMK II, p-JNK, p-p38,ROS,SOD	Oxidative stress, mitochondrial apoptosis	NMDAR-CaMKII-MAPKs	[140]
CUMS male C57BL/6 J mice	XYS0.25 g/kg/d	FLU2.6 mg/kg/d	28 days	↓RAGE	Reduce inflammation	RAGE-mediated inflammatory	[141]
CUMS male C57BL/6 J mice	XYS0.254 g/kg/d	Nec-1(10 mg/kg)	3 weeks	↑IL-1β; ↓RIPK1, RIPK3, MLKL,p-MLKL,IBA1 and Lipocalin-2	Apoptosis of hippocampal neurons, neuroinflammation, and alleviation of microglial activation	RIPK1-RIPK3-MLKL	[142]
CIS male SD rats	XYS3.854 g/kg/day	AG4905 mM65, 66, 2 μL per lateral ventricle	/	↑TNF-α,p-JAK2; ↓Bcl-2	Damage to hippocampal neurons	TNF-α/JAK2-STAT3	[143]
CIS male SD rats	XYS2.22 g/kg/d	FLU2.0 mg/kg/d	21 days	↑cAMP,CREB1,mRNA; ↓PGE2、SP	Reduce inflammatory response and improve the pathological structure of the cerebral cortex	cAMP	[144]
CUMS male SD rats	XYS2.22 g/kg/d	FLU2.0 mg/kg/d	6 weeks	↑caspase-3,Bax; ↓Bcl-2,NR3C1	Reduce neuronal apoptosis	miR-200/NR3C1	[145]
CIS male SD rats	XYS3.854 g/kg/day	saline	14 days	↑BDNF,CRF1	/	CRF1R	[146]
CIS male SD rats	XYS3.854 g/kg/day	FLU1.76 g/kg/d	21 days	↓CORT,NES1,POMC, and MC4R Ob-R,POMC,α-MSH,ARC	Neural pathway	NES1-OT-POMC neural	[147]
CIS male SD rats	XYS3.854 g/kg/day		21 days	/	Improve appetite and weight	leptin-Ob-R-POMC	[148]
CSDS male C57BL/6 J mice	XYS0.3 g/kg/d	FLU3.0 mg/kg/d	1 weeks	↑AdipoR1,p-AMPK,p-ACC	Ameliorate glucose tolerance impairment and increase the level of serum adiponectin	AdipoR1/AMPK/ACC pathway	[149]
CSDS male C57BL/6 J mice	SSa25,50,100 mg/kg/d	FLU20mg/kg/d	10 days	↑Tet1,Notch,DLL3,BDNF; ↓caspase(CASP) 11,CASP-1,NLRP3,IL-1β	Hippocampal nerve damage, increased synapses	tet1/dll3/notch1	[150]
LPS male ICR mice	SSd0.5,1 mg/kg/d	sterile saline	2 weeks	Bcl2; ↓IL-1β,IL-6,TNF-α,LPA1,RhoA,ROCK2,Bax,cleaved-caspase3,p-p38, p-ERK, p-p65and p-IκBα	Inhibit neuronal apoptosis and suppress the production of pro-inflammatory cytokines	LPA1/RhoA/ROCK2	[151]
CRS male SD rats	AF15-30 mg/kg/d	FLU2mg/kg/day	21 days	↑5-HT,5-HIAA,NE,DA,BDNF; ↓NO and cGMP	Neuroprotection, reducing glucocorticoid stress	NO-mediated network	[154]
LPS male SD rats	NBP30mg/kg/d	/	14 days	↑Nrf2,HO-1,NQO-1,pp 65,p-IκB; ↓IL-1β,IL-6,Caspase-3,NF-κB	Neuroinflammation, oxidative stress, neuronal apoptosis	Nrf2 and NF-κB	[155]

## Discussion

This review synthesizes evidence regarding the regulatory effects of XYS and its active compounds on depression-related signaling pathways. The following sections discuss the key findings, limitations, and future directions in a structured manner.

## Summary of key findings

We found that current research primarily focuses on XYS’s ability to reduce inflammatory responses, alleviate hippocampal pathology, inhibit neuronal and mitochondrial apoptosis, suppress oxidative stress, and improve synaptic structure. These effects are largely mediated through the modulation of key pathways, including PI3K/Akt, NLRP3, NF-κB, and MAPK. However, fewer studies have explored the regulation of depression-related pathways by XYS from the perspectives of the HPA axis, neurotransmitter balance, or modulation of gut microbiota.

### Mechanistic insights beyond pathways

Despite this gap, our group and others have demonstrated the antidepressant effects of XYS from broader biological perspectives.

HPA Axis and Neurotransmitters: Our research has shown that XYS modulates the hypothalamic apelin–APJ system, a key regulator of HPA axis activity [156], thereby mitigating depressive symptoms [157]. We have also observed that XYS may exert antidepressant effects by modulating the HPA axis, hippocampal GFAP, and NMDA-type glutamate receptors [21]. As neurotransmitter imbalance is central to the pathogenesis of depression, other studies have shown that XYS regulates Oct1 and Oct3 protein expression in astrocytes, promoting dopamine uptake and enhancing neurotransmitter transport [20].

Gut-Brain Axis: Our previous work demonstrated that XYS improves depression-like behavior by enhancing intestinal barrier function, modulating the expression of tight junction proteins, and regulating gut microbiota and their metabolites such as SCFAs [158, 159]. Additional studies indicate that XYS can affect the “gut–liver–kidney” axis in CUMS rats, thereby alleviating depressive symptoms [44].

### Signaling pathway crosstalk: a key insight into XYS's Mechanism

Signaling pathway research holds promise for drug development by identifying druggable targets, informing screening models, clarifying mechanisms of action, and guiding the development of combination therapies for complex diseases [160]. For example, studies have demonstrated that divergent branches of shared signaling pathways can enhance signal specificity [161], and that positive feedback loops within MAPK cascades can amplify outputs for a greater biological impact [162]. Other reviews, such as those examining the relationship between SOX15 and Wnt signaling, have highlighted the clinical relevance of this cross-regulation for disease prognosis and biomarker identification [163].

A critical integrative insight into XYS’s antidepressant mechanism lies in the intricate cross-talk among the PI3K/Akt, NF-κB, NLRP3, MAPK, and BDNF/CREB pathways, which collectively rebalance neuroinflammation and neuroplasticity. Activation of PI3K/Akt by XYS and its active components (e.g., paeoniflorin, saikosaponin D) not only promotes neuronal survival and synaptic plasticity via downstream mTOR and GSK3β [48, 65] but also inhibits NF-κB signaling by suppressing IκB phosphorylation, thereby reducing pro-inflammatory cytokine release (e.g., IL-1β, TNF-α) that would otherwise exacerbate NLRP3 inflammasome activation [99, 116]. The NLRP3 inflammasome and NF-κB form a feed-forward loop—NF-κB upregulates NLRP3 transcription, while activated NLRP3 amplifies NF-κB-mediated inflammation—both of which are constrained by XYS to alleviate neuroinflammation [98, 105]. MAPK pathways (JNK, ERK, p38) act as key intermediaries: XYS inhibits JNK/p38 activation to reduce neuronal apoptosis and neuroinflammation [134, 135], while enhancing ERK signaling to reinforce BDNF/CREB pathway activation, a core driver of synaptic plasticity and neurotransmitter balance [136, 139]. BDNF/CREB further modulates the network by upregulating PI3K/Akt and suppressing NF-κB/NLRP3 [49, 71], creating a pro-survival, anti-inflammatory feedback loop. Together, XYS orchestrates this interconnected pathway network to break the cycle of neuroinflammation-impaired neuroplasticity, underscoring its multi-target advantage over single-pathway interventions [56, 120].

However, most current studies investigate individual pathways in isolation. Future studies on XYS should prioritize investigating its effects on intersecting and integrative signaling networks, which hold promise for identifying novel druggable targets and guiding combination therapies.

### Current limitations and variability in the evidence base

The strength of the evidence supporting the antidepressant effects of XYS and its active compounds is bolstered by the methodological consistency across the included preclinical studies. A total of 49 studies were included, involving various models of depression. The majority of investigations employed well-validated depression models, such as CUMS, CIS, LPS-induced inflammation, and OB-induced depression. Behavioral assessments—including SPT, FST, OFT, and TST—were consistently applied across studies, enhancing the comparability of outcomes. Moreover, the frequent use of positive controls (e.g., fluoxetine) and dose–response designs further strengthens the internal validity of the findings.

However, several limitations and variabilities exist in the current body of research. Most studies to date have relied on animal models, which differ significantly from human biology. Key interspecies differences exist in HPA axis regulation, gut-brain axis interactions, and drug metabolism [40, 44]. Significant variability exists in dosing regimens (e.g., XYS ranged from 0.93 to 30 g/kg/day) and treatment durations (2–8 weeks), which may influence the translational relevance and dose optimization of XYS. Furthermore, the predominance of male rodents (over 80% of studies) and the lack of comorbid disease models limit the generalizability of the conclusions, particularly given the higher prevalence of depression in females and the high rate of comorbidities in human patients.

### Clinical translation: evidence, challenges, and future imperatives

The clinical evidence base for XYS is substantial, as demonstrated by meta-analyses of 26 randomized controlled trials involving 1,837 patients, which have shown that XYS is non-inferior to SSRIs in treating depression, with a more favorable safety profile [36, 38]. This efficacy is robust across diverse patient populations and study designs. For instance, RCTs indicate that XYS monotherapy significantly reduces HAMD-17 scores in mild to moderate depression, particularly improving psychomotor retardation and rebalancing gene expression and DNA methylation patterns [25]. When used as an adjuvant therapy, MDXY combined with SSRIs like sertraline demonstrates superior response rates and greater reductions in anxiety compared to SSRI monotherapy, with benefits for sleep disturbance and somatic anxiety [33]. This synergistic effect is pronounced in post-stroke depression, where adjunctive XYS therapy achieves a high HAMD score reduction rate. Furthermore, the safety profile of XYS remains favorable across all formulations, with adverse event rates comparable to those of the placebo and no serious adverse events directly linked to treatment. Multicenter trials have confirmed a strong response rate in patients with mild to moderate MDD[25, 26], and significant improvements in post-stroke depression [34]. However, it is important to note contexts where XYS may not be effective, such as in post-COVID-19 depression, highlighting the need for population-specific efficacy assessments [32].

Despite the promising evidence, key translational challenges remain. A primary challenge is formulation standardization, as UPLC-MS has identified 102 bioactive compounds, necessitating batch-to-batch consistency protocols and bioequivalence studies for different preparations like decoctions, pills, and granules [24]. Another challenge is the mechanistic elucidation, which requires human biomarker studies to confirm the modulation of pathways such as PI3K/Akt and NLRP3, as well as gut-brain axis parameters. Although existing clinical evidence of restored serum metabolite levels provides a strong foundation [164], further studies are needed to confirm these findings. Population considerations also present a challenge, as future preclinical models need to adopt sex-balanced designs, construct comorbidity models, and focus on understudied populations, such as the elderly. Meanwhile, clinical research should deliberately stratify analyses to identify the most responsive subgroups.

Looking ahead, future research imperatives should focus on two main areas. First, there is a need for large-scale phase III RCTs with active comparators, long-term follow-up, and subgroup analyses stratified by biomarkers, building on findings that specific symptom clusters respond well to XYS. Second, formulation development must prioritize optimizing active component combinations and developing novel delivery systems capable of penetrating the blood–brain barrier, guided by the consistent efficacy of different modified formulations in clinical trials [165]. This expanded and data-informed translational roadmap bridges the critical gap between the demonstrated effectiveness of XYS and its broader clinical implementation, while clearly outlining the necessary steps to address existing challenges.

## Conclusion and prospects

This review comprehensively summarizes current research on the modulation of depression-related signaling pathways by XYS and its active constituents in animal models. XYS exerts its therapeutic effects through the targeted, multi-mechanism modulation of key molecular pathways, including anti-inflammatory actions, neuronal protection, mitochondrial modulation, and regulation of the gut-brain axis. A promising finding is its potential to modulate crosstalk between critical pathways, such as PI3K/Akt and NF-κB.

Despite promising preclinical findings and existing clinical evidence, several critical limitations must be acknowledged, primarily the reliance on rodent models, variability in experimental designs, and a focus on isolated pathways. Based on our group's experimental evidence, future work will continue to investigate its impact on signaling pathways, particularly the cross-regulation between the PI3K/Akt and NF-κB signaling pathways. Furthermore, building on extensive preclinical findings, future clinical trials are warranted to evaluate the efficacy of XYS in treating human patients with depression, potentially offering more effective and mechanistically informed therapeutic strategies. The integration of precision medicine approaches with traditional formula optimization positions XYS as a promising candidate for the development of next-generation antidepressants.

## Supplementary Information

Supplementary file 1.

Supplementary file 2.

## Data Availability

Not applicable.

## Abbreviations

5-HT: Serotonin
16SrRNA: 16S ribosomal RNA
1H NMR: Proton nuclear magnetic resonance
Abcc5: ATP binding cassette subfamily C member 5
ACLK: ATP citrate lyase kinase
AGE-RAGE: Advanced glycation end-products
AKT1: Protein kinase B alpha
Akt: Protein kinase B
BDI: Beck depression inventory
BDNF: Brain derived neurotrophic factor
bid: Bis in die
CA1: Hippocampal cornu ammonis 1
CA-3: Cornu ammonis 3
ceRNA: Competing endogenous RNA
Cfb: Complement factor B
CUMS: Chronic unpredictable mild stress
DElncRNAs: Differentially expressed lncRNAs
DEcircRNAs: : Differentially expressed circRNAs
DEmiRNAs: Differentially expressed miRNAs
DEmRNAs: Differentially expressed mRNAs
FAAH: Fatty acid amide hydrolase
GLDH: Glutamate dehydrogenase
Gpc3: Glypican-3
GO: Gene ontology
GPX3mRNA: Glutathione peroxidase 3 messenger RNA
GOT: Glutamic-oxalacetic transaminase
HAMD: Hamilton depression rating scale
HRSD: Hamilton rating scale for depression
HIF-1: Hypoxia-Inducible Factor1
HO-1: Heme oxygenase-1
HPA: Hypothalamus pituitary adrenal
IL-1β: Interleukin-1β
IL-6: Interleukin-6
Keap1: Kelch-like ech-associated protein 1
LC/MS: Liquid chromatography/Mass spectrometry
Lmx1b: Lim homeobox transcription factor 1 beta
MAP2: Microtubule-associated protein 2
MAPK: Mitogen-Activated Protein Kinase
MADD: Mixed anxiety-depressive disorder
MDD: Major depressive disorder
MDXYS: Modified Xiao-Yao-San
mTOR: Mammalian target of rapamycin
MRCC-V: Mixed-radical chain chlorination of vinyl vhloride
NE: Norepinephrine
NF-κB: Nuclear factor-κB
NFKB1: Nuclear Factor Kappa-B Subunit 1
NLRP3: NOD-like receptor family, pyrin domain containing 3
NQO1: NAD(P)H quinone oxidoreductase 1
Nrf2: Nuclear factor erythroid 2-related factor 2
OB: Olfactory bulbectomy
PC: Prothrombin complex
PDH: Pyruvate dehydrogenase
PI3K: Phosphatidylinositol 3 Kinase
PIK3R1: Phosphoinositide-3-Kinase Regulatory Subunit 1
PSD: Post-stroke depression
qd: Quaque die
RCTs: Randomized controlled trials
ROS: Reactive oxygen species
SDH: Synchronous digital hierarchy
SDS: Self-rating depression scale
SIRM: Stable isotope-resolved metabolomics
SSRIs: Selective serotonin reuptake inhibitors
TCA: Tricarboxylic acid
TCM: Traditional Chinese medicine
TEM: Transmission electron microscopy
tid: Ter in die
TNF-α: Tumor necrosis factor-α
trkB: Tropomyosin receptor kinase B
WGBS: Whole genome bisulfite sequencing
XYS: Xiao-Yao-San
XYW: Xiaoyao Pills
