# Supplementary material for: From classical Chinese formula to modern mechanism: how Xiao-Yao-San modulates key signaling pathways in depression
Source: Chin Med. 2026 Jan 15;21:39. doi: 10.1186/s13020-025-01315-7 (PMC12805791; doi:10.1186/s13020-025-01315-7)
Supplement: Supplementary file 1 [file 13020_2025_1315_MOESM1_ESM.pdf]

Better Papers Faster™

Language Editing Certification

To whom it may concern:

This manuscript, entitled “From Classical Chinese Formula to Modern Mechanism: How Xiao-Yao-San Modulates Key Signaling Pathways in Depression”, has been professionally edited for English usage, grammar, spelling, and punctuation by a native English speaker and a skilled professional editor. Therefore, we would appreciate if any comments regarding language are as specific as possible.

Genesis Technology Communication (Beijing), Co., Ltd.

E-mail: sabine@manuscriptrevisions.com

Nov 15, 2025

Better Papers Faster
