# Supplementary figures and images for: From classical Chinese formula to modern mechanism: how Xiao-Yao-San modulates key signaling pathways in depression
Source: Chin Med. 2026 Jan 15;21:39. doi: 10.1186/s13020-025-01315-7 (PMC12805791; doi:10.1186/s13020-025-01315-7)

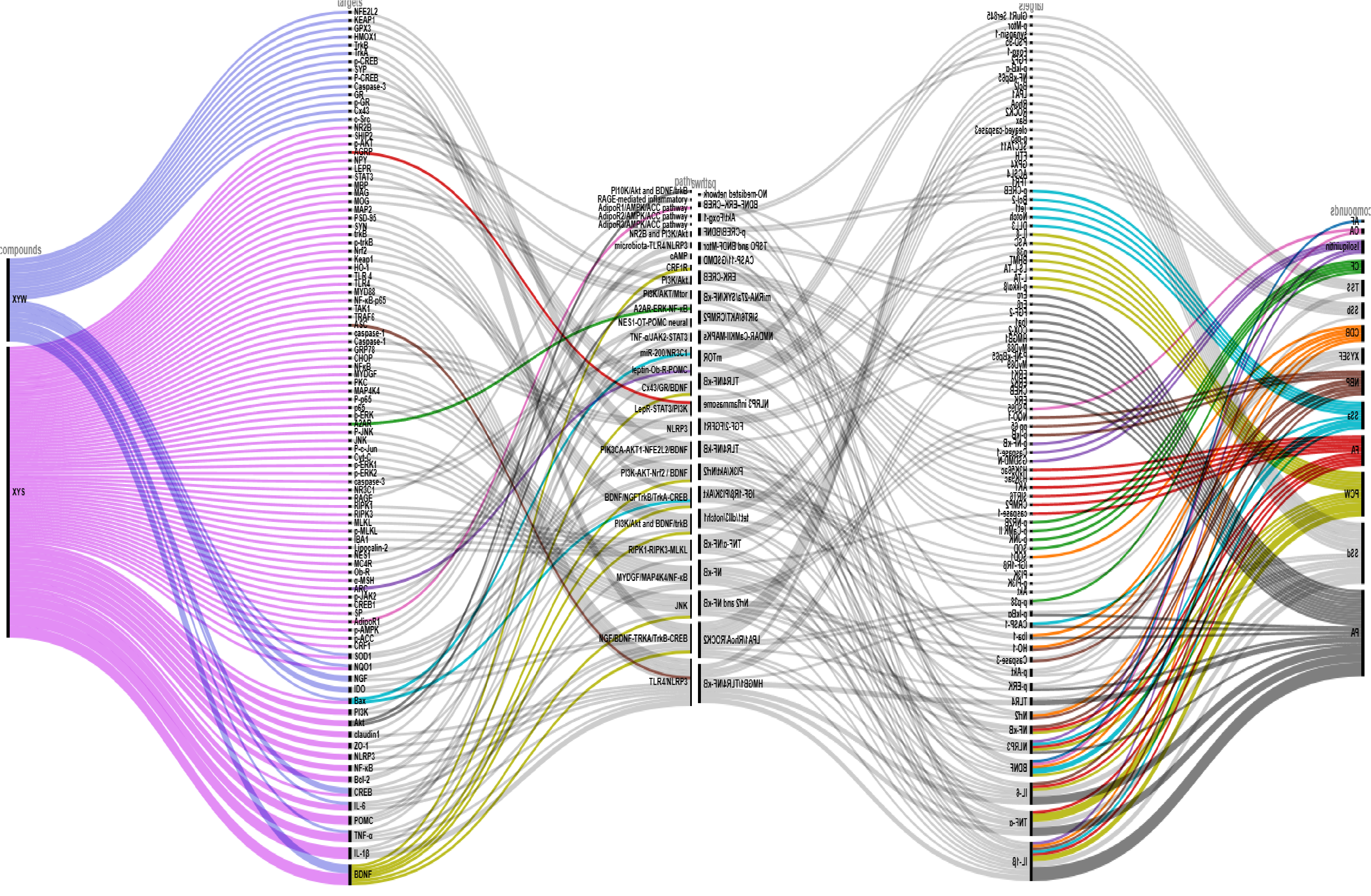

Supplement: Supplementary file 2 [file 13020_2025_1315_MOESM2_ESM.png]
